# Inhibition of Thioredoxin Reductase Activity and Oxidation of Cellular Thiols by Antimicrobial Agent, 2-Bromo-2-nitro-1,3-propanediol, Causes Oxidative Stress and Cell Death in Cultured Noncancer and Cancer Cells

**DOI:** 10.3390/biology14050509

**Published:** 2025-05-06

**Authors:** Chao Jiang, Gary Krzyzanowski, Dinesh S. Chandel, Wesley A. Tom, Nirmalee Fernando, Appolinaire Olou, M. Rohan Fernando

**Affiliations:** Molecular Diagnostic Research Laboratory, Center for Sensory Neuroscience, Boys Town National Research Hospital, Omaha, NE 68131, USA

**Keywords:** thioredoxin reductase, 2-bromo-2-nitro-1,3-propanediol, thioredoxin reductase inhibition, apoptosis, cancer cells, cell death, necrosis, oxidative stress

## Abstract

Background: The thioredoxin system (TrxS) is crucial for maintaining redox balance by regulating cellular thiol levels and is involved in various biological processes, including cancer progression. Thioredoxin reductase (TrxR), a key component of TrxS, reduces oxidized thioredoxin (Trx) using NADPH. This study investigates the inhibitory effects of 2-bromo-2-nitro-1,3-propanediol (Bronopol, BP), a preservative, on TrxR activity and its impact on cellular thiols and cell viability. Methods: Purified recombinant TrxR and noncancer and cancer cells were treated with different concentrations of BP and TrxR activity measured. BP-treated cells were examined for effects of BP on total cellular thiol level and GSH/GSSG ratio. Results: BP effectively inhibited TrxR in a dose-dependent manner, an effect that was reversible with dithiothreitol (DTT). BP treatment significantly reduced total thiol levels, decreased the GSH/GSSG ratio, and increased reactive oxygen species (ROS) in cells. Additionally, BP decreased cell viability and induced apoptosis, as indicated by morphological changes and increased c-fos mRNA expression. Conclusions: These findings highlight BP’s potential as a TrxR inhibitor and its cytotoxicity toward both noncancer and cancer cells. The observed effects—TrxR inhibition, thiol oxidation, GSH/GSSG imbalance, and ROS accumulation—may underlie BP’s cytotoxicity. Further research is needed to explore the precise molecular mechanisms by which BP exerts these effects.

## Simple Summary

Our cells rely on a system called the thioredoxin system (TrxS) to maintain balance and protect against damage. A key player in this system is an enzyme called thioredoxin reductase (TrxR), which helps keep cells healthy by regulating certain protective molecules. This study looked at how a common preservative called Bronopol (BP) affects TrxR and overall cell health. Researchers tested BP on both normal and cancer cells to see if it interferes with TrxR activity. They found that BP blocked TrxR in a way that depended on the dose used, but this effect could be reversed with another compound. BP also reduced important protective molecules in cells, increased harmful reactive oxygen species (ROS), and led to cell damage. In both normal and cancer cells, BP treatment resulted in decreased cell survival and signs of programmed cell death (apoptosis). These results suggest that BP has the potential to disrupt essential cell functions and may contribute to cell death by increasing stress and damage within cells. More research is needed to fully understand how BP causes these effects and whether it could have implications for human health.

## 1. Introduction

**Figure 1:**
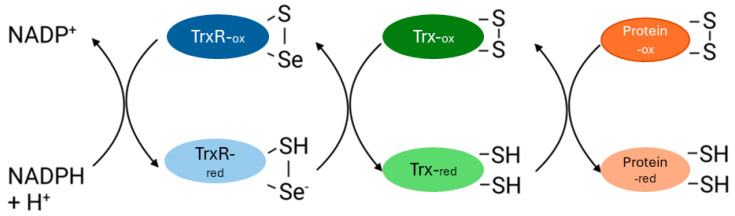
Graphic representation of the mammalian TrxS. Reduced Trx catalyzes the reduction of oxidized proteins into reduced proteins. During this process Trx becomes oxidized. Oxidized Trx is converted to reduce Trx by TrxR and NADPH.

The mammalian thioredoxin system (TrxS) encompasses the interplay of several essential components, namely, thioredoxin reductase (TrxR), thioredoxin (Trx), and nicotinamide adenine dinucleotide phosphate hydrogen (NADPH), which is a crucial cofactor that protects cells from oxidative damage. Trx, a 12 KDa protein, possesses an active site with two redox-active cysteine residues [1]. TrxR, on the other hand, is a selenoprotein with a molecular mass of approximately 55 kDa, characterized by an active site featuring a selenocysteine and a cysteine residue [2]. The oxidized Trx is reduced by TrxR, and TrxR is then reduced by NADPH (Figure 1). Ubiquitously expressed, TrxS plays a crucial role in maintaining cellular and extracellular redox homeostasis [3]. One of its primary functions is to sustain a low redox potential and a high level of free thiols in cells, in conjunction with cellular glutathione (GSH). Oxidized GSH is reduced by glutathione reductase [4,5]. Additionally, Trx participates in DNA biosynthesis by serving as a two-electron donor for the enzyme ribonucleotide reductase [6]. The TrxS acts as a hydride donor for methionine sulfoxide reductase, an enzyme crucial for reducing methionine sulfoxide residues in proteins damaged due to oxidation [7]. In mammalian cells, TrxS assumes a pivotal role in the redox regulation of transcription factors such as NFkB and AP-1 [8,9]. Furthermore, TrxS is known to play a crucial role in the regeneration of oxidatively damaged proteins, thereby protecting mammalian cells [10].

Elevated levels of Trx and TrxR have been observed in several types of cancers [11,12]. The tumor microenvironment, characterized by oxidative stress or hypoxia, can induce increased levels of Trx and TrxR in cancerous cells [12]. The augmented expression of Trx and TrxR may contribute to cancer progression and drug resistance by suppressing oxidative stress and hypoxic conditions [13,14,15]. It has been postulated that elevated levels of TrxR could disrupt cell cycle regulation through excessive activation of growth factors and inhibition of apoptosis, ultimately leading to the malignant transformation of cells [16]. Numerous investigations have explored the inhibitory effects of specific chemical agents on TrxR in cancer cells, highlighting TrxR as a promising target for cancer therapy [17,18,19].

The compound, 2-bromo-2-nitro-1,3-propanediol, also known as Bronopol (BP), is a broad-spectrum antimicrobial agent widely employed in various industries, including pharmaceutical and cosmetic sectors, as a preservative [20,21]. This compound demonstrates high water solubility and exerts antimicrobial activity at low concentrations. It has been reported that BP possesses the ability to oxidize thiol groups in proteins [22,23]. Mammalian TrxR and Trx possess catalytic thiol groups within their active sites, which are essential for their biological activity. Oxidation of these catalytic thiol groups renders TrxR and Trx biologically inactive [1,2]. In this study, we investigate the potential of BP to inhibit TrxR and induce cell death, comparing cultured noncancer and cancer cells.

## 2. Materials and Methods

### 2.1. Cell Culture and Materials

The HeLa cell line, human pancreatic cancer cell line AsPC-1, and human ovarian epithelial cancer cell line SKOV-3 were purchased from the American Type Culture Collection (ATCC). Human pancreatic duct epithelial cell line H6C7 was purchased from Kerafast, Inc. (Boston, MA, USA). Ovarian cancer cell line, OVCAR-5, was purchased from Cell Biolabs Inc. (San Diego, CA, USA). Immortalized human ovarian surface epithelial cell line, HOSE, is a gift from Dr. John Davis’s laboratory, department of Obstetrics and gynecology, University of Nebraska Medical Center, Omaha, NE, USA. HeLa cells, OVCAR-5, SKOV-3, and AsPC-1 cells were grown in DMEM medium supplemented with 10% fetal calf serum and penicillin/streptomycin (100 µg/mL), in a humid atmosphere of 5% CO_2_ at 37 °C. H6C7 cells were grown in DermaLife Basal Medium from ATCC (with growth factor kit) supplemented with 10% fetal calf serum and penicillin/streptomycin (100 µg/mL), in a humid atmosphere of 5% CO_2_ at 37 °C. HOSE cells were grown in Medium 199 and MCDB medium 1:1 ratio supplemented with 10% fetal calf serum and penicillin/streptomycin (100 µg/mL), in a humid atmosphere of 5% CO_2_ at 37 °C. The compound 2-Bromo-2-nitro-1,3-propanediol (BP) and dithiothreitol (DTT) were purchased from Thermo Scientific (Waltham, MA, USA). Human recombinant TrxR was purchased from Cayman Chemicals (Ann Arbor, MI, USA). Illustra MicroSpin Sephadex™ G-25 columns containing DNA grade F resin was purchased from GE Healthcare (Chicago, IL, USA).

### 2.2. Detection of Free Formaldehyde in Cell Culture Medium Containing BP

Quantofix^®^ formaldehyde test strips (Macherey-Nagel, GmbH & Co. KG, Düren, Germany) were used to detect formaldehyde in BP-treated cell culture medium following the manufacturer’s recommended protocol. Results are based on comparison of the test strip color with color fields on the container of the strips exactly 60 s after immersion in the cell culture medium.

### 2.3. Inhibition of TR Activity

BP stock solutions were prepared by dissolving the compound in PBS to achieve the desired concentrations. HeLa, HOSE, and OVCAR-5 cell lysates were prepared by homogenizing cell pellet in cold homogenizing buffer (50 mM potassium phosphate, pH 7.4 containing 1 mM EDTA) and then centrifuging at 10,000× *g* for 15 min at 4 °C. The supernatant was used for TrxR activity measurements. TrxR activity, using recombinant human TrxR (150 µg/mL) and cell lysates from HeLa, HOSE, and OVCAR-5 cells (1000 µg/mL total protein), was measured by a TrxR assay kit following the manufacturer’s instructions (Cayman Chemicals, Ann Arbor, MI, USA). The TrxR used in this experiment was in a reduced state (in the presence of NADPH). TrxR activity was calculated using the formula given in the instruction manual. To detect the TrxR inhibition of BP, the desired concentrations of the compound were added to the reaction mixture, and TrxR activity was measured.

### 2.4. Reactivation of BP-Inhibited TR, by DTT

Human recombinant TrxR (150 µg/mL) was treated with BP (final concentration of 50 µM) and incubated at room temperature for 5 min. The reaction mixture was aliquoted, and TrxR activity was measured in the presence or absence of DTT (1000 µM) treatment for 10 min. After incubation, the mixture was passed through a Sephadex™ G-25 column to remove excess DTT, and BP and TrxR activities were compared.

### 2.5. Quantification of Total Thiols in BP-Treated and Untreated Cells

Total thiols in HOSE, HeLa, OVCAR-5, and AsPC1 cells were quantified using a previously described method [24]. Noncancer and cancer cells were grown to achieve 70–80% confluence and treated with 100 µM BP in serum-free medium for 3 h. Then, cells were collected by trypsinization and lysed in RIPA buffer for 30 min on ice with occasional vortexing. Total thiol levels were determined by DTNB (5,5-dithio-bis-(2-nitrobenzoic acid) method. Cell lysate (10 µL) was mixed with 90 µL DTNB reagent (1 mM DTNB in 6 M guanidine hydrochloride, pH 8.0), incubated at room temperature for 5 min, and 412 nm absorbance was measured using a spectrophotometer. Total thiol level was calculated using a GSH standard curve.

### 2.6. Detection of Intracellular ROS Concentration Using Fluorescence Dye DCFH-DA

Cells grown in 12-well plates were treated with 50 µM BP in serum-free medium for 3 h. After removal of the medium, serum-free medium containing 10 µM oxidative stress indicator DCFH-DA was added onto cells and incubated for 30 min at 37 °C in the dark. The cells were observed under an inverted fluorescent microscope and photographed.

### 2.7. Cell Viability by MTT Assay

To investigate the cytotoxic effects of BP, the cells (70–80% confluent) were treated with various concentrations of the compound for 24 h. To assess cell viability, a modified MTT (3-(4,5-dimethylthiazol-2-yl)-2,5-diphenyltetrazolium bromide) assay was performed as previously described [25]. In brief, following BP treatment, 10 µL of MTT reagent (5 mg/mL in PBS) was added directly to each well containing the treated cells. After a 4 h incubation period, the medium was removed and 100 µL of DMSO was added. Cell viability was assayed by the absorbance at 570 nm using Molecular Device’s SpectraMax microplate reader (Sunnyvale, CA, USA). The absorbance values obtained from the MTT assay were used to determine the relative cell viability of the treated HeLa cells compared to control cells.

### 2.8. Detection of Apoptosis by Hoechst 33342 Staining Method and Annexin V and Propidium Iodide (PI) Double Staining Method

Cells at 70–80% confluency were treated with 50 µM BP for a duration of 24 h and gently washed with PBS (1ⅹ) to remove any residual compound or culture media. To assess apoptosis, the cells were stained with Hoechst 33342 reagent, a fluorescent DNA-binding dye commonly used to visualize nuclear morphological changes associated with apoptosis [26], for 5 min at room temperature. Subsequently, the cells were washed three times with PBS to remove any unbound dye. Finally, the stained cells were observed and photographed using a ZEISS fluorescence microscope (Carl Zeiss Microscopy GmbH, Jena, Germany) to detect apoptotic cell morphological changes such as nuclear condensation and fragmentation.

To detect apoptosis using FITC-Annexin V and PI double staining, the Annexin V and PI Apoptosis Kit (cat# A025; ABP Biosciences, Beltsville, MD, USA) was used following the manufacturer’s instruction. Treated and untreated cells were stained with FITC-Annexin V and PI following the manufacturer’s instructions, and cells were observed and photographed using a ZEISS fluorescence microscope (Carl Zeiss Microscopy GmbH, Jena, Germany) to detect apoptotic and necrotic cells.

### 2.9. Detection of c-fos mRNA by Droplet Digital PCR (ddPCR)

Total RNA was extracted from BP-treated and untreated noncancer and cancer cells using the GeneJet RNA purification kit (Life Technologies Corp., Carlsbad, CA, USA). The nucleotide sequences for primers and probe for c-fos have been previously described [27], and were purchased from Integrated DNA Technologies (Coralville, IA, USA). Complementary DNA (cDNA) was synthesized using iScript™ Reverse Transcription Supermix for RT-qPCR (Bio-Rad, Hercules, CA, USA). Reverse transcription (RT)-ddPCR was performed on the QX200 Droplet Digital PCR system (Bio-Rad, Hercules, CA, USA) using the ddPCR^TM^ Kit for probes as previously described [28].

### 2.10. Detection of ATP Concentration in Treated and Untreated HeLa Cells

HeLa cells were plated in a 96-well plate and incubated to achieve a 70–80% confluence. These cells were treated with increasing doses of BP (12.5, 25, 50, 75, or 100 µM) over a 24 h duration. Intracellular ATP concentration was determined using the ATPlite luminescence ATP detection assay system (PerkinElmer, Shelton, CT, USA, cat No. 6016943) following the manufacturer’s recommended protocol.

### 2.11. TrxR Activity

TrxR activity was measured by 5, 5′-dithiobis (2-nitrobenzoic) acid (DTNB) reduction assay. DTNB assay is widely used to measure the TrxR activity in biological samples in vitro [24,29,30]. TrxR catalyzes the reduction of DTNB with NADPH to 5-thio-2-nitrobenzoic acid (TNB2-). TNB2 generates a strong yellow color with maximum absorbance at 412 nm. TrxR activity was detected by the DTNB method, using a commercially available thioredoxin reductase colorimetric assay kit (Cayman Chemicals, Ann Arbor, MI, USA). TrxR activity was calculated using the formula given in the kit instruction manual.

### 2.12. Detection of Cellular Total Glutathione (GSH) and GSH/GSSG) Ratio

Cells were seeded into a clear-bottom 96-well microtiter plate at a density of 8000 cells/well in complete DMEM medium and incubated for 24 h. Cell culture medium was replaced with HBBS (Sigma-Aldrich, St. Louis, MO, USA) to minimize background fluorescence interference. Cells were treated with either 50 µM or 100 µM BP for 2 h at 37 °C. Cell lysates were prepared and analyzed for glutathione using the Promega (Madison, WI, USA) GSH/GSSG-Glo assay following the manufacture’s recommended protocol. GSH and GSSG concentrations were measured following the manufacturer’s instruction. The luminescent signal was measured using Molecular Device’s SpectraMax microplate reader (Sunnyvale, CA, USA). GSH concentration was determined using a GSH standard curve. The GSH/GSSG ratio was calculated as [(net total glutathione RLU − net GSSG RLU)/(net GSSG RLU)] × 2, where RLU is relative light units.

### 2.13. Statistical Analysis

GraphPad Quick Calcs *t*-test calculator online software was used for statistical analysis (http://www.graphpad.com/quickcalcs/ttest1.cfm accessed on 21 April 2024). Analysis was performed using an unpaired, two-tailed Student’s *t*-test, and *p* < 0.05 was considered statistically significant.

## 3. Results

### 3.1. BP Reversibly Inhibits TrxR Enzyme Activity in In Vitro and in Cultured Cells

**Figure 2:**
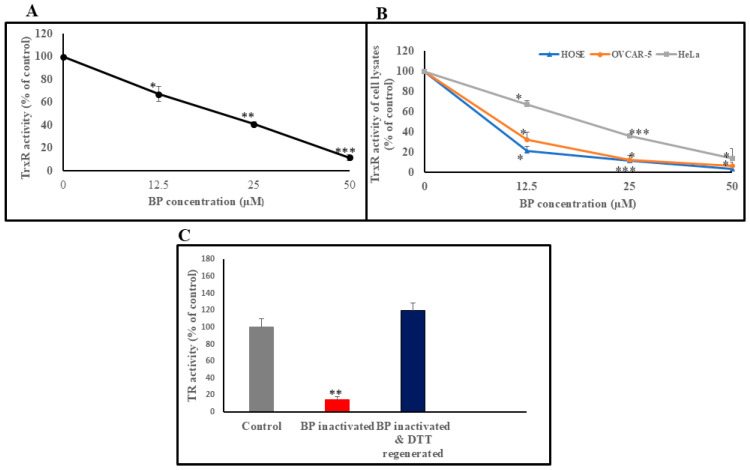
Effect of BP on human recombinant TrxR (**A**) compared with TrxR activities in HOSE, HeLa, and OVCAR-5 cell lysates (**B**). TrxR activity expressed as percentage of the untreated/unaffected control enzyme or cell lysates. Increasing concentrations of BP (12.5, 25, and 50 µM) were used in the reaction mixture. The IC50 value for human recombinant TrxR was 21 µM. The IC50 values for HOSE, HeLa, and OVCAR-5 cell lysates were 8.5, 20.5, and 10 µM, respectively. Panel (**C**) shows the reversible nature of BP-mediated TrxR inhibition using human recombinant TrxR. BP-inactivated TrxR was treated with DTT, and TrxR activity was measured as described in methods. The results are based on three independent experiments. Error bars indicate SD. * *p* < 0.05, ** *p* < 0.001, *** *p* < 0.0001.

To evaluate the inhibitory effect of BP on TrxR, human recombinant TrxR was incubated with increasing doses of BP, as outlined in the methods section. As shown, TrxR inhibition due to BP was observed as a decrease in TrxR activity (Figure 2A). These results indicate potent inhibitory effect of BP on human recombinant TrxR. The IC50 value for BP in this assay was calculated to be 21 µM. We also investigated the ability of BP to inhibit TrxR activity in cell lysates obtained from three cell lines (HOSE cells, immortalized noncancer cell line, and two cancer cell lines: HeLa and OVCAR-5). Cells treated with increasing concentrations of BP led to reductions in TrxR activity in HeLa, HOSE, and OVCAR-5 cells (Figure 2B). The IC50 values for BP in these cells were 20.5 µM (HeLa), 8.5 µM (HOSE), and 10 µM (OVCAR-5), respectively. The results above strongly indicate that BP targets TrxR activity.

### 3.2. Reactivation of BP-Inhibited TrxR, by DTT

To test the hypothesis that TrxR inhibition by BP is mediated through reversible thiol oxidation, we investigated the potential of a strong reducing agent, DTT, for a possible reversal of BP-mediated TrxR inhibition. When human recombinant TrxR was treated with 50 µM BP, there was an 85% reduction in TrxR activity. However, when the BP-inactivated TrxR was treated with DTT a complete recovery of TrxR activity was observed (Figure 2C). This indicates that the BP-mediated TrxR inhibition is reversible (by DTT) and that BP acts via thiol modification.

### 3.3. Effect of BP on Total Thiol Concentration in Cells

**Figure 3:**
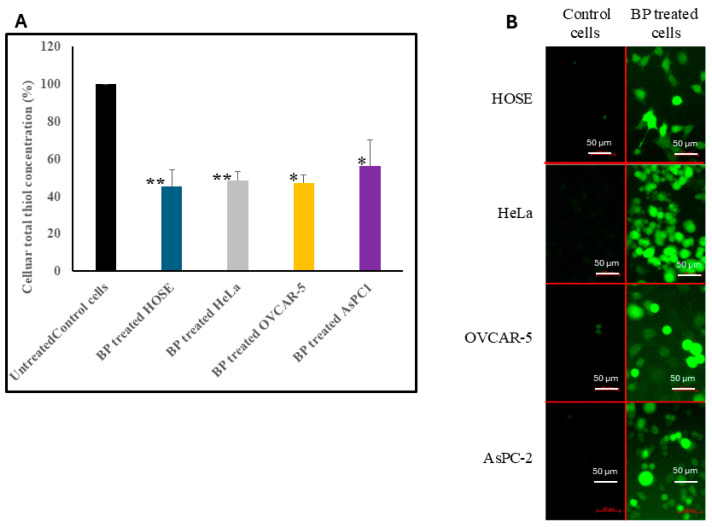
Effect of BP on total thiol concentration in HOSE, HeLa, OVCAR-5, and AsPC-1 cells. (**A**) Total thiol concentrations compared in BP-treated and untreated cell lysates (see methods). (**B**) Effect on ROS concentration in cells treated with 50 µM BP for 3 h. The cellular ROS levels were detected by DCFH-DA fluorescence dye, and images were acquired using an inverted fluorescence microscope. Error bars indicate SD. * *p* < 0.05, ** *p* < 0.001: scale bars indicate 50 µm.

TrxR is essential for maintaining thiol balance in cells. Inhibition of TrxR may lead to accumulation of oxidized thiols, thus decreasing the total reduced thiol concentration in cells. To test this hypothesis, we measured the total thiol concentrations in BP-treated and untreated cells. HOSE, HeLa, OVCAR-5, and AsPC1 cells treated with 100 µM BP for 3 h resulted in a 57.4%, 46.1%, 43.6%, and 47.5% drop in total thiol concentrations, respectively (Figure 3A). These results demonstrate the ability of BP to oxidize cellular thiols by inhibiting TrxR and TrxS, and, hence, cause oxidative stress in cells.

### 3.4. Effect of BP on Intracellular ROS Concentrations

The effect of BP on cellular ROS concentration was examined using DCFH-DA fluorescence dye. One of the major functions of the TrxS is to maintain the intracellular redox balance and defend the cells against oxidative stress. Therefore, the inhibition of TrxR by BP may lead to the accumulation of intracellular ROS. Hence, we determined the ROS level in cells before and after BP treatment. DCFH-DA is capable of rapidly diffusing into cells, and inside cells it is converted to nonfluorescent 20′, 70′-dichlorodihydrofluorescin by cellular esterase. Nonfluorescent 20′, 70′-dichlorodihydrofluorescin reacts with cellular ROS to give highly fluorescent 20′, 70′-dichlorofluorescein. Control cells treated only with DCFH-DA showed minimal or no signals, whereas cells treated with BP resulted in a strong green fluorescence, indicating increased concentrations of ROS compared to untreated cells (Figure 3B). We also quantified intracellular ROS levels by measuring fluorescence intensity using Fiji ImageJ software version 2.16.0. In all four cell lines, BP-treated cells exhibited significantly higher fluorescence intensities compared to untreated controls (Supplementary Materials, Figure S1).

### 3.5. Effect of BP on Cell Viability

**Figure 4:**
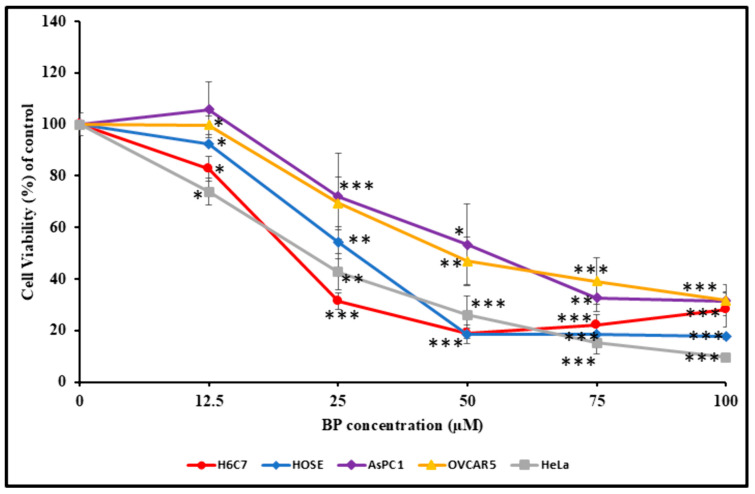
Effect of BP on H6C7, HOSE, AsPC-1, OVCAR-5, and HeLa cell viability. Cells treated with increasing concentrations of BP for 24 h show a dose-dependent decrease in cell viability. The IC50 values for H6C7, HOSE, AsPC-1, OVCAR-5, and HeLa cells were 21, 28, 56.25, 47, and 22, µM respectively. Error bars indicate SD. * *p* < 0.05, ** *p* < 0.001, *** *p* < 0.0001.

Given the numerous important roles of the thioredoxin system (TrxS) in cells, namely, in redox regulation of transcription [8,9] and protection against oxidative damage [7,10], we investigated the impact of BP-mediated TrxR inhibition on cell viability. Five cell lines (HeLa, HOSE, OVCAR-5, AsPC-1, and H6C7) were treated with four different concentrations of BP (12.5, 25, 50, and 100 µM) for 24 h. Assessment of cell viability by MTT assay, as described in the methods section, indicates that BP inhibits cell viability in dose- and cell-type-dependent manners (Figure 4). Treating HOSE and H6C7 cells (normal, noncancerous) with BP resulted in a decrease in cell viability (Figure 3), with IC50 values at 28 and 21 µM, respectively. Treating HeLa, OVCAR-5, and AsPC-1 cells with BP resulted in a decrease in cell viability (Figure 4), with IC50 values at 22, 47, and 56.25 µM, respectively. The results above suggest that BP can induce cell death in cancer as well as noncancer cells.

### 3.6. Effect of BP on Cellular ATP Level

**Figure 5:**
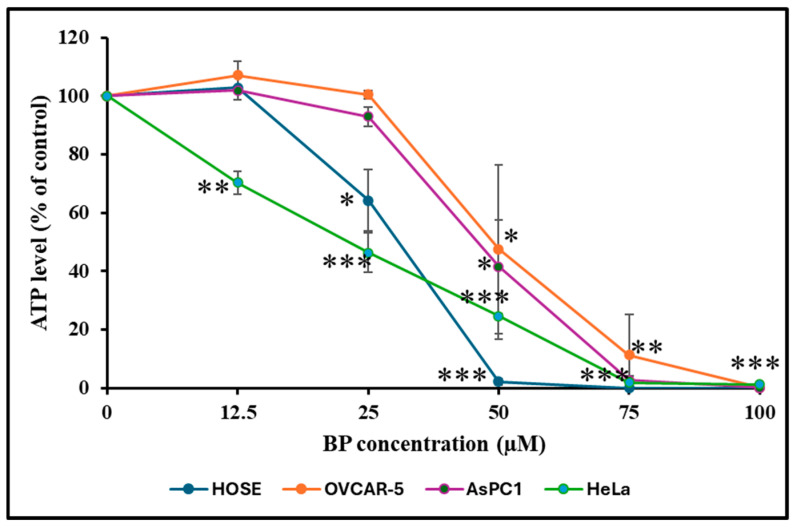
Effect of BP on ATP concentration in HOSE, HeLa, AsPC-1, and OVCAR-5 cells. Cells were treated with increasing concentrations of BP for 24 h, and cellular ATP levels were determined as described in the methods. Except for an initial increase in ATP levels in cells treated with a low (starting) BP concentration (12.5 µM), there was a drop in cellular ATP levels overall with increasing BP dose, compared to untreated control. Error bars indicate SD. * *p* < 0.05, ** *p* < 0.001, *** *p* < 0.0001.

Declining cellular ATP concentration is a marker for cell death. Thus, we compared the ATP concentration in cells treated with increasing concentrations of BP at 37 °C for 24 h. As depicted in Figure 5, treating HOSE cells with 12.5 µM BP increased cellular ATP concentration by 2.8%; however, increasing BP doses of 25, 50, 75, and 100 µM resulted in reduced cellular ATP concentrations. Treating HeLa cells with different BP concentrations also reduced cellular ATP levels. Treating OVCAR-5 cells with 12.5 µM BP increased ATP by 7%, and there was no change when BP concentration was increased to 25 µM. OVCAR-5 cells exposed to BP concentrations of 50, 75, and 100 µM showed reduced cellular ATP levels. AsPC-1 cells exposed to 12.5 µM BP showed a 1.9% increase in ATP levels. BP concentrations at 25, 50, 75, and 100 µM resulted in overall reduced cellular ATP levels (Figure 5).

### 3.7. BP-Induced Apoptosis

**Figure 6:**
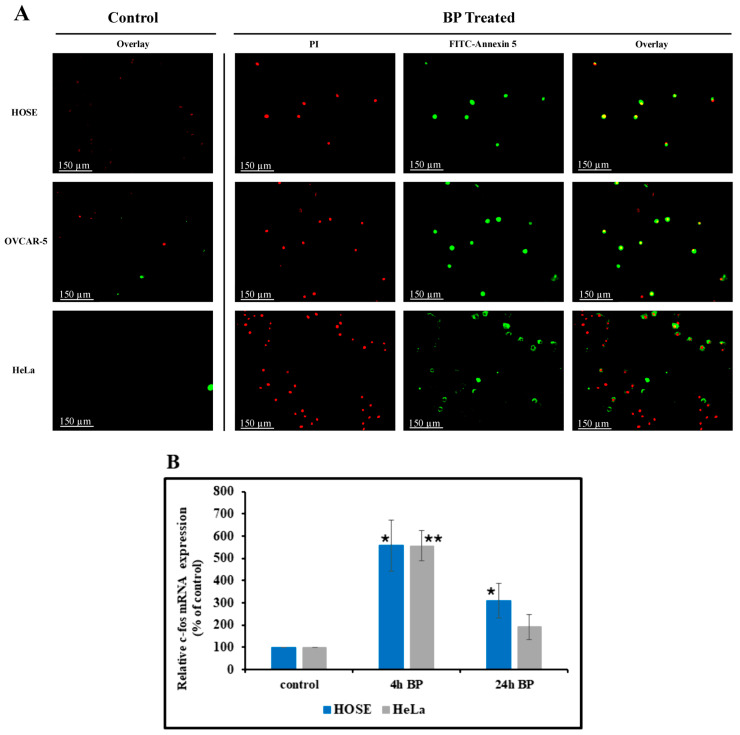
Effect of BP on apoptosis (**A**) and cellular c-fos mRNA level (**B**). (**A**) Compared to un-treated (control), BP-treated (50 µM, 24 h) cells stained with FITC-Annexin V and PI stain show green fluorescence (for FITC-Annexin V) and a red fluorescence (Texas Red dye representing PI-stained cell nucleus). Cells stained in green are early apoptotic cells. Cells with red color show necrosis. Merged images show the cells with both green and red color, which are late-stage apoptotic cells. (**B**) Both HOSE and HeLa cells show a transient increase in cellular c-fos mRNA levels detected after 4 h of BP treatment. This increase started to drop after 24 h in both cell lines; however, it remained significant in HOSE cells, compared to untreated control. Total RNA was extracted from treated and untreated control cells, and c-fos mRNA expression levels were determined using ddPCR (see methods). Error bars indicate SD. * *p* < 0.05, ** *p* < 0.001.

The effect of BP on apoptosis was studied using two methods: the Annexin-V-FITC/PI double staining method and Hoechst 33342. Figure 6A shows the results obtained from the Annexin V/PI staining and the fluorescence microscopy analysis method. The treated cell population showed a significant increase in cells with bright green membrane staining (Annexin V positive) compared to the control group, indicating a higher proportion of early apoptotic cells. Additionally, a small population of cells displayed both green membrane and red nuclear staining, suggesting the presence of late apoptotic or necrotic cells.

Apoptosis was also detected using the Hoechst 33342 staining method. To assess apoptotic changes, both BP-treated and untreated cells were stained with Hoechst 33342 and observed under a fluorescence microscope. While untreated cells showed no effects, BP-treated cells revealed distinct nuclear fragmentation and condensation (Supplementary Materials Figure S2), suggestive of apoptotic cell death. These findings suggest that BP might influence the apoptotic process, as evidenced by the specific morphological changes observed in the treated cells. These findings suggest that BP might influence the apoptotic process, as evidenced by the pronounced morphological changes observed in the treated cells.

### 3.8. Effect of BP on Cellular c-fos mRNA Level

To evaluate apoptotic cell death, we measured c-fos mRNA induction in BP-treated HOSE and HeLa cells. c-fos has been previously shown to mediate cell death by apoptosis [31]. HOSE and HeLa cells were either treated or untreated with BP (50 µM) for 4 or 24 h, and the c-fos mRNA copy number was determined using droplet digital PCR (ddPCR). As shown in Figure 6B, there is a statistically significant transient increase in c-fos mRNA levels at 4 h post BP treatment. This mRNA increase started to drop after 24 h in both cell lines; however, there was a statistically significant increase in HOSE cells compared to untreated control.

### 3.9. Detection of Total GSH and GSH/GSSG Ratio

**Figure 7:**
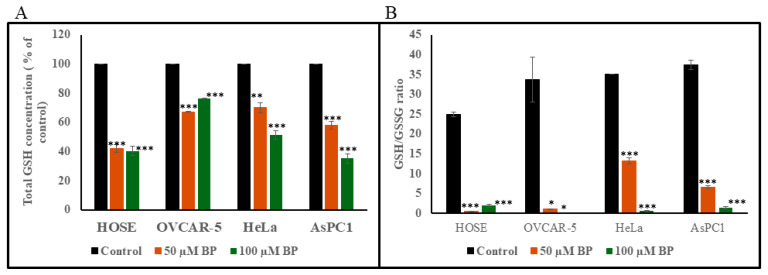
Effect of BP on cellular total GSH (**A**) and GSH/GSSG ratio (**B**). Cells treated with BP (50 µM or 100 µM) for 2 h at 37 °C showed a significant decrease in GSH levels, thereby causing a significant drop in GSH/GSSG ratios. Error bars indicate SD. * *p* < 0.05, ** *p* < 0.001, *** *p* < 0.0001.

Figure 7A shows the effect of 50 and 100 µM BP on total GSH level in HOSE, OVCAR-5, HeLa, and AsPC1 cells. Exposure of cells at above BP concentrations for 2 h caused a statistically significant decrease in total GSH level in all cell lines tested. GSH/GSSG ratio also dropped significantly in all cell lines (Figure 7B).

### 3.10. Detection of Free Formaldehyde in Cell Culture Medium Containing BP

BP is known as a slow releaser of small amounts of formaldehyde. Therefore, we tested formaldehyde concentration in cell culture medium treated with BP using Quantofix^®^ formaldehyde test strips. After cells were treated with BP, formaldehyde concentration was determined in cell culture medium at 4 and 24 h, and it was found that formaldehyde is undetectable in cell culture medium with BP (Supplementary Materials Figure S3).

## 4. Discussion

TrxS is an important redox regulator in mammalian cells. It protects cells from oxidative stress by maintaining the balance of the thiol-disulfide redox status [32]. Therefore, changes in TrxS expression level may affect the cellular physiology. There are numerous reports that show that TrxS is overexpressed in cancer cells [14,15,33]. Also, it has been suggested that elevated levels of TrxS could disrupt cell cycle regulation through excessive activation of growth factors and inhibition of apoptosis, leading to the malignant transformation of cells [14]. Hence TrxS has emerged as a potential molecular target for cancer therapy.

It is well established that both Trx and TrxR have catalytic/thiol groups in their active sites, and oxidation/modification of these thiol groups can inactivate them [32,34]. BP is a well-known antimicrobial agent widely used in pharmaceutical and cosmetic industries as a preservative [20,21]. This compound has the potential to oxidize/modify protein thiol groups [22,23]. Hence, we initiated this study to evaluate its potential as a TrxR inhibitor.

To gain further insight into the nature of BP action, we assessed if this BP-mediated TrxR inhibition was reversible. Our results demonstrate that the treatment of BP-inactivated TrxR with the reducing agent DTT resulted in a complete recovery of TrxR activity. Oxidation of thiol groups in TrxR by BP is a covalent modification, as resulting products are either disulfide or sulfenic acid. Therefore, regenerating thiol groups by DTT should involve breakdown of the covalent bonds. Therefore, the inhibition of TrxR by BP is a reversible covalent inhibition [35]. This suggests that BP inhibits TrxR via oxidation/modification of the thiol groups on TrxR—a phenomenon that is reversible by a strong reducing agent DTT—which provides a clue of a flexible regulation of TrxR activity and a promising avenue for intervention strategies in diseases induced due to an aberrant TrxR activity.

It has been shown that cellular TrxR inhibition causes accumulation of ROS in cells, which in turn leads to a significant decrease in cellular thiol concentration [24]. Our results show that the inhibition of TrxR by BP causes significant decreases in cellular thiol concentration and significantly increases ROS levels in HOSE (noncancer cell line), HeLa, OVCAR-5, and AsPC-2 (cancer cell lines).

Indeed, the impact of TrxR inhibition by BP on cell viability indicates that BP can target both noncancer and cancer cells. Our results demonstrated that the IC50 values for noncancer cell lines, HOSE and H6C7, and cancer cell line, HeLa, were similar, while OVCAR-5 and AsPC-1 cells showed high IC50 values, indicating that these cell lines are somewhat resistant to oxidative stress caused by BP.

To elucidate the mechanism of cell death induced by BP, we explored the apoptotic pathway. Transient induction of c-fos is implicated in the activation of apoptosis in various cell types [36,37]. Studies have shown that c-fos induction is necessary and precedes the apoptotic chromatin condensation and oligonucleosomal DNA fragmentation [38]. Our findings suggest that BP-induced cell death occurs primarily through apoptosis. We observed a transient increase in c-fos mRNA expression, a marker of apoptotic cell death, following BP treatment in HOSE and HeLa cells. However, the c-fos mRNA levels dropped after 24 h, indicating a transient apoptotic response. This temporal pattern suggests that BP treatment triggers an early apoptotic event, which subsides over time.

To further confirm the apoptotic effect of BP, we examined the morphological changes in HOSE, HeLa, OVCAR-5, and SKOV-3 cells post BP treatment. BP-treated cells exhibited distinct nuclear fragmentation and condensation, as evidenced by Hoechst 33342 staining. In contrast, untreated cells showed no such changes. The presence of fragmented and condensed nuclei strongly supports the notion that BP induces apoptosis in HeLa cells. It has been reported that programmed cell death process requires ATP [39,40]. In previous studies, a transient increase in ATP concentration during early stage of apoptosis has been observed. However, if cell death occurs via necrosis, cellular ATP concentration shows a drastic decline [41]. Therefore, we compared the ATP concentrations in BP-treated cells to those of the untreated control. Our results also indicate that cells could experience an apoptotic process even at a low BP concentration (12.5 µM). However, HeLa cells showed a significant drop in ATP level at the same BP levels, indicating that necrosis may be the prominent death process. Cells treated with 50, 75, and 100 µM BP for 24 h showed a statistically significant drop in ATP concentrations. Therefore, exposure of these cells to high BP concentrations may induce cell death via necrotic process.

As apoptosis is triggered by numerous factors, the precise molecular mechanisms of BP-mediated TrxR inhibition should be further investigated. Cellular redox homeostasis plays a very important role in maintaining the balance between cell survival and death. One of the key players in maintaining cellular redox homeostasis is TrxS. The TrxS is critical for defense against oxidative stress, and regulation of apoptosis. Recent studies have demonstrated that ROS and the resulting oxidative stress play an important role in apoptosis [42]. ROS are generated in cells as byproducts of normal metabolic processes and are rapidly converted to H_2_O_2_. Because the accumulation of H_2_O_2_ is toxic to cells, it is detoxified by several enzymatic systems. One such enzyme is peroxiredoxin, which neutralizes H_2_O_2_ using reduced thioredoxin (Trx) as a cofactor. Inhibition of TrxR by BP disrupts the regeneration of reduced Trx, thereby impairing peroxiredoxin’s ability to detoxify H_2_O_2_ and contributing to ROS accumulation. Another important enzyme involved in H_2_O_2_ detoxification is glutathione peroxidase, which requires reduced glutathione (GSH) to function. In our study, we demonstrated that BP treatment drastically reduced intracellular GSH levels, which may impair glutathione peroxidase activity and further contribute to the accumulation of ROS in BP-treated cells. The consequent accumulation of ROS leads to oxidative stress, damaging cellular proteins, lipids, and DNA. Oxidative damage to these biomolecules triggers the intrinsic apoptotic pathway, involving mitochondrial dysfunction and the release of cytochrome c. High ROS levels can also lead to the activation of proapoptotic signaling pathways, such as JNK and p38 MAPK, which further enhance apoptotic processes [43]. The inhibition of TrxR, oxidation of cellular thiols, and reduction in GSH/GSSG ratio promote the activation of proapoptotic proteins such as Bax and Bak, which translocate to the mitochondria and form pores in the mitochondrial membrane [44]. This pore formation facilitates the release of cytochrome c, initiating the apoptotic cascade. Simultaneously, TrxR inhibition diminishes the activity of antiapoptotic proteins like Bcl-2 and Bcl-xL, shifting the balance in favor of apoptosis [45]. TrxR is involved in the regulation of proteins like ribonucleotide reductase, which is essential for DNA synthesis and repair. Inhibition of TrxR results in impaired DNA repair mechanisms, increasing the likelihood of DNA-damage-induced apoptosis. Reduced Trx is required for S-nitrosation of procaspase-3 and the inhibition of apoptosis in Jurkat cells [46]. Inhibition of TrxR may result in low levels of reduced Trx, which may lead to the inhibition of S-nitrosation of procaspase-3 and, hence, the induction of apoptosis.

We also studied the total GSH and GSH/GSSG ratio in cancer cells (HeLa, OVCAR-5, and AsPC1) and noncancer cells (HOSE), showing that both total GSH and GSH/GSSG ratio drops significantly in BP-treated cells. At present, we do not know whether this drop in GSH and GSH/GSSG ratio is due to increased ROS concentration resulted from TrxR inhibition or inhibition of glutathione reductase by BP. This should remain a focus of future mechanistic studies.

BP in solution is known to slowly release small amounts of formaldehyde. A study previously reported that formaldehyde (>1.0 mM) can induce apoptosis in cancer as well as noncancer cells. The same study showed that 0.1 mM formaldehyde enhanced cell proliferation [47]. Therefore, we investigated formaldehyde levels in BP-treated cell culture medium. Our results show that formaldehyde remained undetectable in BP-treated cell culture medium after 4 and 24 h. This provides further evidence that BP-induced apoptosis is not mediated via formaldehyde.

In our study, we demonstrated that BP possesses potent cytotoxic effects, inducing both apoptosis and necrosis in HeLa, OVCAR-5, and AsPC1 cancer cells. However, it is important to acknowledge that BP also exerts similar cytotoxic effects on noncancerous cells (HOSE), which presents a significant challenge for its direct therapeutic application. Despite this limitation, it would be premature to dismiss BP’s potential role in cancer treatment. Many effective chemotherapeutic agents are inherently toxic to both malignant and normal cells; the key to their clinical utility lies in selective targeting strategies that minimize off-target toxicity. Modern advancements in drug delivery systems, including nanoparticle-based carriers, liposomes, antibody–drug conjugates, and ligand-directed delivery mechanisms, offer promising avenues to enhance the selective delivery of cytotoxic agents like BP to tumor tissues while sparing healthy cells. By incorporating BP into such targeted delivery platforms, it may be possible to exploit its potent anticancer properties while significantly reducing its adverse effects on normal tissues. Additionally, tumor microenvironments often exhibit distinct physiological characteristics—such as enhanced permeability, acidic pH, and overexpression of specific receptors—that can be strategically utilized to improve the selective accumulation and uptake of BP-loaded delivery systems in cancer cells. Therefore, while the broad cytotoxicity of BP presents an obstacle, it also provides an opportunity to explore innovative delivery approaches that could unlock its therapeutic potential in oncology.

There are several limitations to our study that warrant consideration. One notable limitation is the lack of detailed investigation into the precise mechanisms by which BP inhibits TrxR. While our findings suggest that BP exerts an inhibitory effect on TrxR, the exact molecular pathways and interactions underlying this effect remain unclear. Future studies should, therefore, aim to elucidate the specific mechanisms by which BP targets and modulates TrxR activity.

Another limitation of our work is its narrow focus on the TrxS, which, although one of the major redox regulatory systems within cells, represents only a part of the broader cellular redox landscape. Mammalian cells possess several other crucial redox regulatory systems that maintain redox homeostasis and protect against oxidative stress. These include the glutathione system—comprising GSH and glutathione reductase (GR); the glutaredoxin system—consisting of glutaredoxin, GSH, and GR; and additional antioxidant systems such as glutathione peroxidase, the peroxiredoxin system, and catalase. The potential impact of BP on these systems has not been explored in this study, representing a significant gap in our understanding. To gain a more comprehensive view of BP’s cellular effects, future research should investigate its influence on these additional redox regulatory networks.

Moreover, it is important to recognize that the proper functioning of all redox systems is heavily dependent on the availability of reducing equivalents, particularly NADPH. NADPH is primarily generated through the citric acid cycle and the hexose monophosphate (HMP) shunt (also known as the pentose phosphate pathway). Disruption of NADPH generation can profoundly affect the activity of redox regulatory enzymes and the cell’s ability to counter oxidative stress. Therefore, it would be valuable for future investigations to examine the effects of BP on these two major NADPH-producing pathways.

While our study provides insights into the cytotoxic and antitumor effects of BP through detailed in vitro molecular and cellular analyses, there are some limitations that should be acknowledged. Notably, we did not include in vivo animal model studies in this investigation. As such, the systemic effects, pharmacokinetics, biodistribution, and potential toxicity of BP in a physiological context remain to be determined.

Finally, a significant limitation of our current study is the absence of in vivo tumor-bearing animal model experiments. Although we have demonstrated BP’s mechanistic effects using in vitro cellular models, further studies in tumor-bearing animal models are needed to evaluate its pharmacokinetics, biodistribution, systemic toxicity, and overall therapeutic potential. Inclusion of such data would help bridge the gap between preclinical findings and potential clinical applications.

## 5. Conclusions

In summary, to elucidate the mechanisms underlying the cytotoxic and antitumor properties of BP, we conducted a series of in vitro and cellular assays. We first demonstrated that BP inactivates thioredoxin reductase (TrxR) both in vitro and in vivo. Using purified TrxR enzyme, we confirmed BP-mediated inhibition in a cell-free system. In cellular models, treatment of both cancerous and noncancerous cell lines with BP at various time points resulted in a significant reduction in TrxR activity. This inhibition was reversible, as co-treatment with the reducing agent dithiothreitol (DTT) restored enzyme activity, suggesting that BP acts via reversible redox modification.

To assess the downstream effects of TrxR inhibition, we measured cell viability following BP exposure and observed a dose-dependent decrease in viability across both cancerous and normal cell lines. We next evaluated oxidative stress markers. BP treatment significantly elevated intracellular reactive oxygen species (ROS), accompanied by decreased protein thiol content, reduced total glutathione (GSH), and a lower GSH/GSSG ratio—indicators consistent with oxidative stress-mediated cytotoxicity.

To determine the mode of cell death, we employed Annexin V-FITC/PI double staining and Hoechst 33342 nuclear staining. These assays confirmed that BP induces apoptosis in both cell types. Supporting this, droplet digital PCR analysis revealed a transient increase in c-fos mRNA expression following BP treatment, consistent with apoptotic signaling activation.

To further distinguish between apoptotic and necrotic responses, intracellular ATP levels were quantified. At low BP concentrations, cells exhibited a transient rise in ATP levels, characteristic of apoptosis. In contrast, higher BP concentrations led to ATP depletion, suggesting a shift toward necrotic cell death.

Our study provides important insights into the anticancer potential of BP, demonstrating its ability to induce both apoptosis and necrosis in cultured cancer cells. At the same time, we acknowledge the significant limitation posed by its cytotoxic effects on noncancerous cells, which raises concerns about its safety profile in clinical applications. Nevertheless, this limitation should not overshadow the therapeutic promise that BP holds. As with many established chemotherapeutic agents, the challenge lies not solely in the inherent toxicity of the compound but in the ability to harness and direct its effects selectively toward malignant cells while minimizing collateral damage to healthy tissues. In conclusion, while the broad cytotoxicity of BP presents a challenge, it simultaneously offers a valuable opportunity to explore innovative, targeted therapeutic strategies that could ultimately position BP as a viable anticancer agent within the context of modern oncology.

## 6. Future Directions

Advances in targeted drug delivery technologies offer a promising strategy to address this challenge. The development of nanoparticle-based formulations, liposomal carriers, antibody–drug conjugates, and receptor-mediated delivery systems provides opportunities to enhance the tumor-specific delivery of BP, potentially reducing systemic toxicity and improving therapeutic efficacy. Furthermore, the unique characteristics of the tumor microenvironment—including enhanced vascular permeability, altered pH, and differential expression of surface receptors—can be exploited to achieve preferential accumulation and activation of BP within tumor tissues.

Looking ahead, future studies should focus on elucidating the precise molecular mechanisms by which BP induces cell death, as well as evaluating its effects on additional redox regulatory systems beyond the thioredoxin system. Investigating the impact of BP on NADPH-generating pathways and other antioxidant defense systems will be crucial for developing a comprehensive understanding of its mode of action. Moreover, preclinical studies incorporating BP into advanced, tumor-targeted delivery platforms are warranted to evaluate its therapeutic potential in vivo and to determine its safety and efficacy profiles in more physiologically relevant models.

## Data Availability

The relevant raw datasets for specific experiments of interest are available from the authors upon request.

## Supplementary Materials

The following supporting information can be downloaded at: https://www.mdpi.com/article/10.3390/biology14050509/s1, Figure S1: Effect of BP on cellular ROS level; Figure S2: Effect of BP on apoptosis; Figure S3: Detection of formaldehyde in BP treated cell culture medium.

## Abbreviations

The following abbreviations are used in this manuscript:

TrxS	Thioredoxin system
Trx	Thioredoxin
TrxR	Thioredoxin reductase
BP	Bronopol
