# Supplementary material for: Inhibition of Thioredoxin Reductase Activity and Oxidation of Cellular Thiols by Antimicrobial Agent, 2-Bromo-2-nitro-1,3-propanediol, Causes Oxidative Stress and Cell Death in Cultured Noncancer and Cancer Cells
Source: Biology (Basel). 2025 May 6;14(5):509. doi: 10.3390/biology14050509 (PMC12108914; doi:10.3390/biology14050509)
Supplement: Supplementary file 1 [file biology-14-00509-s001.zip › biology-3582563-supplementary.pdf]

Supplementary data section

Figure S1Effect of BP on cellular ROS level

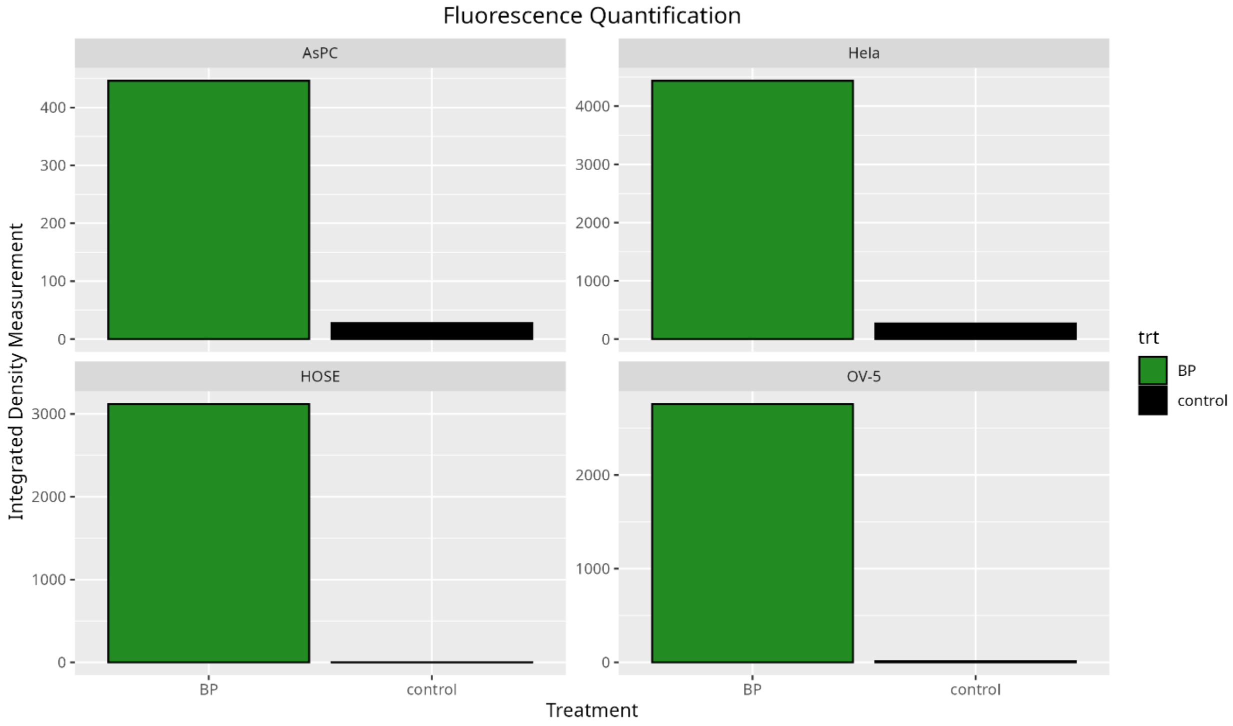

Figure S1. Area-integrated fluorescence intensity was measured from microscopy images of BP-treated and untreated cells using ImageJ software. The resulting fluorescence intensity values were visualized using ggplot2 in R. In the graphs, green bars represent BP-treated cells, while black bars represent untreated controls. The plots are faceted by cell line: AsPC1, HeLa, HOSE, and OVCAR-5.

Figure S2: Effect of BP on apoptosis

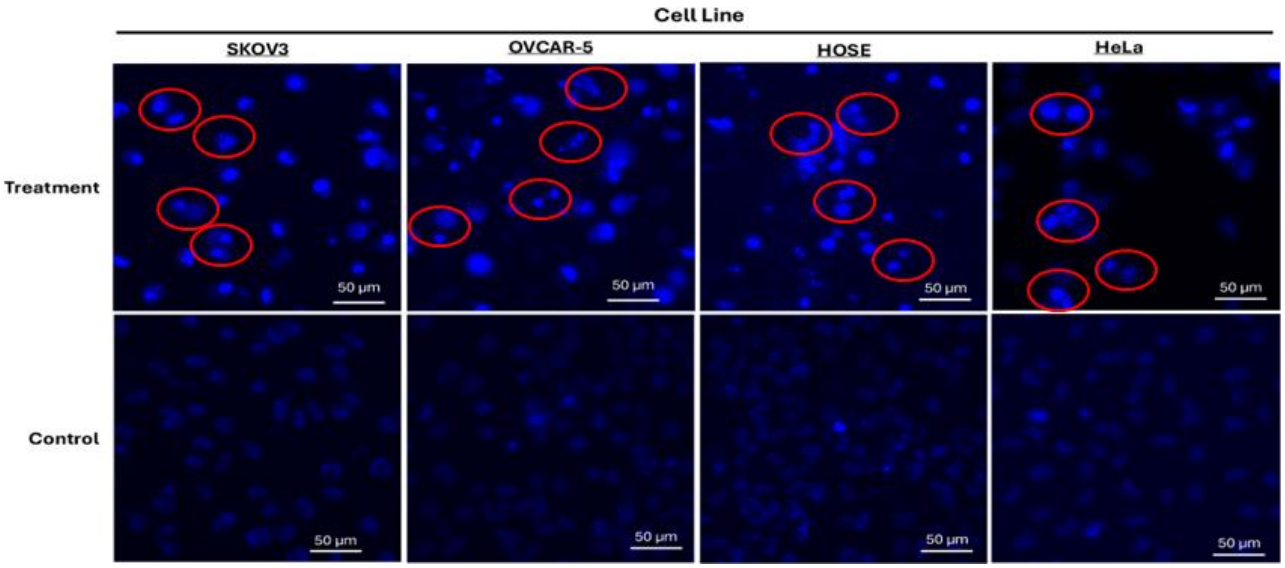

Figure S2: Effect of BP on apoptosis. Compared to untreated (control), BP treated (50 µM, 24 hours) cells stained with Hoechst 33342 nuclear stain show typical apoptotic nuclear morphological changes. The red circles indicate apoptotic cells, which exhibited highly condensed and fragmented nuclear morphologies. Scale bars indicate 50 µm.

Figure S3: Detection of formaldehyde in BP treated cell culture medium.

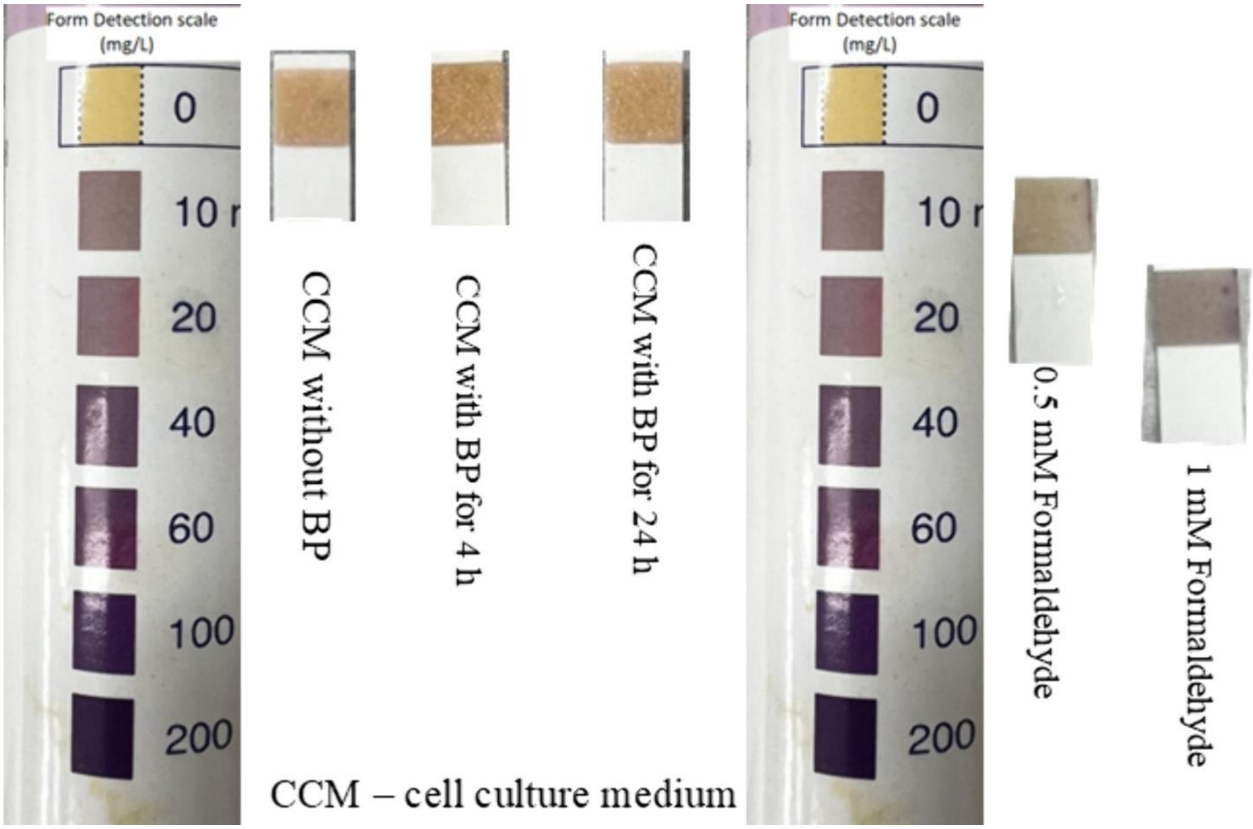

Figure S3. Detection of formaldehyde in BP treated cell culture medium. Quantofix® Colorimetric test strips were used to detect formaldehyde in BP treated or untreated cell culture media. Colors matching on the detection scale (0.0 mg/L) show no traces of formaldehyde detected in culture medium tested after 4 and 24 hours of BP-treatment. For comparison, the standard controls (0.5mM and 1.0 mM) were also matched to color scale corresponding to 10mg/L and 20 mg/L formaldehyde levels, respectively.
